# Time- and temperature-dependent Pentraxin 3 stability in serum and bronchoalveolar lavage fluid samples

**DOI:** 10.1093/mmy/myaf057

**Published:** 2025-06-27

**Authors:** Radim Dobiáš, Valeria Skopelidou, Andrea Langer Sermeño, Jan Strakoš, Dominika Luptáková, Hana Tomášková, Milan Raška, Jozef Škarda, Denisa Bázsóová, Vladimír Havlíček

**Affiliations:** Department of Bacteriology and Mycology, National Reference Laboratory for Mycological Diagnostics, Public Health Institute in Ostrava, Ostrava, Czech Republic; Institute of Laboratory Medicine, Faculty of Medicine, University of Ostrava, Ostrava, Czech Republic; Institute of Laboratory Medicine, Faculty of Medicine, University of Ostrava, Ostrava, Czech Republic; Institute of Molecular and Clinical Pathology and Medical Genetics, University Hospital Ostrava, Ostrava, Czech Republic; Institute of Molecular and Clinical Pathology and Medical Genetics, Faculty of Medicine, University of Ostrava, Ostrava, Czech Republic; Institute of Laboratory Medicine, Faculty of Medicine, University of Ostrava, Ostrava, Czech Republic; Institute of Laboratory Medicine, Faculty of Medicine, University of Ostrava, Ostrava, Czech Republic; Institute of Microbiology of the Czech Academy of Sciences, Prague, Czech Republic; Biomedicine Research Centre of the Slovak Academy of Sciences, Institute of Virology, Bratislava, Slovakia; Department of Bacteriology and Mycology, National Reference Laboratory for Mycological Diagnostics, Public Health Institute in Ostrava, Ostrava, Czech Republic; Institute of Laboratory Medicine, Faculty of Medicine, University of Ostrava, Ostrava, Czech Republic; Department of Immunology, Faculty of Medicine and Dentistry, Palacký University and University Hospital, Olomouc, Czech Republic; Institute of Molecular and Clinical Pathology and Medical Genetics, University Hospital Ostrava, Ostrava, Czech Republic; Institute of Molecular and Clinical Pathology and Medical Genetics, Faculty of Medicine, University of Ostrava, Ostrava, Czech Republic; Department of Bacteriology and Mycology, National Reference Laboratory for Mycological Diagnostics, Public Health Institute in Ostrava, Ostrava, Czech Republic; Institute of Microbiology of the Czech Academy of Sciences, Prague, Czech Republic; Department of Analytical Chemistry, Faculty of Science, Palacký University, Olomouc, Czech Republic

**Keywords:** Pentraxin 3, biomarker, stability, Aspergillosis, serum, bronchoalveolar lavage fluid, storage, enzyme immunoassay, preservation

## Abstract

Pentraxin 3 (Ptx3) is an acute-phase protein that specifically targets fungal galactosaminogalactan and has been proposed as a promising biomarker for invasive fungal infections. However, its stability in clinical samples over time has not yet been established. This study aimed to evaluate the stability of Ptx3 in serum and bronchoalveolar lavage fluid (BALF) samples during mid- and long-term storage. A total of 44 serum and 52 BALF samples were examined or re-examined for Ptx3 concentrations using enzyme immunoassay in pooled and individual sample formats. Samples were stored at −80°C, −20°C, and +37°C for periods ranging from 0 to 56 months. Statistical analyses included a paired two-sample Wilcoxon signed-rank test, analysis of variance, Bonferroni test, and linear regression analysis. Ptx3 remained highly stable in serum and BALF samples for up to 8 months at −20°C, with variations ranging from −1.8% to +2.8%. Long-term stability was observed at −80°C for 48 months, followed by a slow decline in Ptx3 levels. In contrast, storage at +37°C resulted in rapid degradation, with a 36.5%–60.7% increase or a 92.9%–97% decrease in Ptx3 levels in serum and BALF, respectively. These findings confirm that Ptx3 is a stable and reliable biomarker for invasive fungal infections when appropriate storage conditions are maintained.

## Introduction

Invasive fungal infections (IFIs) are a significant cause of morbidity and mortality, particularly in immunocompromised patients. Among these, invasive aspergillosis is the most prevalent and lethal type. Early diagnosis is critical for improving patient outcome. Conventional diagnostic modalities, including culture, histopathology, and radiological imaging, often lack sensitivity and specificity, resulting in delayed diagnosis and treatment. Pentraxin 3 (Ptx3) is an acute-phase protein that participates in the innate immune response to various infections and inflammatory stimuli.^1^ It belongs to the pentraxin family, together with C-reactive protein (CRP) and the serum amyloid P component. Unlike CRP produced by the liver, Ptx3 is produced/secreted directly at the site of infection or inflammation by a range of cell types, including dendritic cells, macrophages, neutrophils, and endothelial cells, in response to inflammatory signals such as interleukin-1 and tumor necrosis factor.^2^ Ptx3 is a pattern-recognition molecule that senses and binds to pathogens, further enhancing their removal by complement-dependent immune mechanisms.^3^

It has been shown that levels of Ptx3 are highly elevated in patients with IFIs, particularly in those with invasive aspergillosis.^4^ A previous investigation conducted by our research group demonstrated that bronchoalveolar lavage fluid (BALF) Ptx3 levels were significantly elevated in patients with invasive aspergillosis compared to those in patients with bacterial infections or colonization by non-*Aspergillus* species. These results suggest that Ptx3 may serve as a potential biomarker for the early detection of infections, showing greater sensitivity in fungal infections than in bacterial infections. It may also serve as a complementary biomarker to distinguish fungal from bacterial infections in BALF samples.^5^ Conversely, it is important to note that the precise role of Ptx3 remains unexplored. According to a study by Li et al. in 2019,^6^ plasma Ptx3 levels were elevated in non-neutropenic patients with pulmonary aspergillosis compared to those in healthy individuals. Most false-positive results occurred in patients with coexisting infections, such as bacterial pneumonia, where sputum cultures revealed the presence of *Pseudomonas aeruginosa* and *Klebsiella pneumoniae*.^6^

In patients with sepsis and septic shock, Ptx3 acts as an independent predictor of mortality, enabling disease severity classification and prediction of 90-day mortality based on the latest Sepsis 3.0 definitions.^7^ Ptx3 levels were significantly increased in non-survivors compared to survivors of sepsis.^8^ Furthermore, Ptx3 has been identified as an unfavorable prognostic marker.^9^ In recent studies, Ptx3 has been presented as a biomarker with potential utility in a range of diseases, including cardiovascular diseases, lung cancer, recurrent pregnancy loss, neonatal sepsis, atherosclerotic heart disease, acne vulgaris, and diabetic nephropathy.^10^ Ptx3 expression is induced in response to inflammatory signals and tissue damage, suggesting its role as an indicator of the inflammatory response and innate immunity.^11^ While the diagnostic and prognostic value of Ptx3 has been highlighted, with its levels changing in response to disease states^8^ in prospective and/or retrospective investigations,^12,13^ the literature does not explicitly address the stability of Ptx3 levels over extended periods or under varying storage conditions.

The primary objective of this study was to evaluate the stability of human Ptx3 stored at −20°C, −80°C, and +37°C in serum and BALF samples to evaluate Ptx3 reliability in both study designs.

## Materials and methods

### Pentraxin 3 assay

The concentration of Ptx3 was measured using an enzymatic immunoassay (EIA) kit (BioVendor; Brno, Czech Republic). The performance of Ptx3 EIA was evaluated according to the manufacturer’s guidelines provided by BioVendor. To ensure accuracy, serum and BALF samples were thawed immediately before testing at each measurement time point. The detection limit of the assay was 22 pg/ml, and the calibration curve points ranged from 78 to 5000 pg/ml.

### Prospective cohort, real-time evaluation

**Figure 1.**
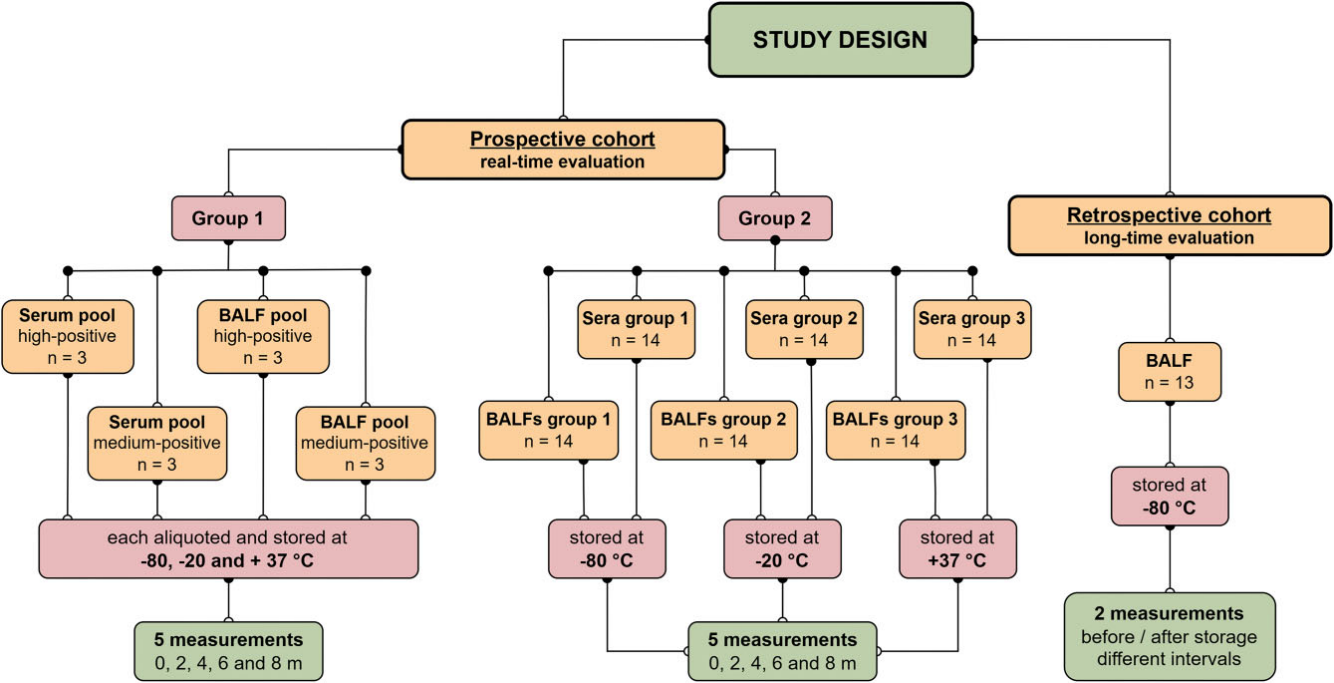
Summarizes the study’s overall design as well as the individual cohorts of the analyzed samples and the method of their investigation. Prospective Group 1 consisted solely of samples from a pool with a medium or high Ptx3 concentration (both for serum and BALF specimens). Group 2 included individual serum and BALF samples that were separated into subsets based on the storage temperature. Samples from both prospective groups were measured five times: 0 m (T0), or the first measurement of the given sample prior to storage at the assigned temperature; 2 m, 4 m, 6 m, and 8 m—T1–T4, or further consecutive measurements of individual samples after previous thawing. The retrospective cohort included BALF samples stored at −80°C. Only two measurements were performed, before freezing and after varying periods of storage.

The patients participated in a prospective study (https://classic.clinicaltrials.gov/ct2/show/NCT05860387), and their samples were separated into two groups. Group 1 represented four distinct pools comprising two sera and two BALFs from three independent donors, each with either medium-positive (∼3000 pg/ml) or high-positive (∼5000 pg/ml) Ptx3 levels (Fig. 1). The medium-positive pool contained specimens with Ptx3 concentrations ranging from 3001 to 3056 pg/ml (mean: 3021 pg/ml), whereas the high-positive pool contained specimens with Ptx3 concentrations ranging from 4956 to 5089 pg/ml (mean: 5012 pg/ml).

The second group, Group 2, consisted of 42 serum and BALF samples divided into three equal cohorts that were used for storage at three different temperatures. These specimens were selected based on the results of Ptx3 testing using the Ptx3 EIA kit performed by the Public Health Institute in Ostrava, a National Reference Laboratory for Mycology Diagnostics, between May 31 and July 25, 2023.

All cohorts of samples were divided into five aliquots, each used for a specific period of storage (Fig. 1). All specimens fulfilled the manufacturer’s original testing standards, which included storage at 2°C–8°C for up to 48 h and at −20°C for more than 48 h prior to the initial clinical testing (T0). A single technologist performed testing using a single lot (E22-036S11) from the BioVendor Ptx3 EIA kit. Each aliquot from both specimen groups underwent a single freeze-thaw cycle, with the longest test date set at 8 months. Measurements were taken at regular 2-month intervals. There were five measurements in total, including the initial evaluation (T0), evaluation after 2 months (T1), 4 months (T2), 6 months (T3), and the final one after 8 months of storage (T4).

The criteria for determining whether Ptx3 levels are “stable” or “unstable” under various conditions were explicitly defined, with a maximum deviation of ±5% from the baseline at T0 for Group 1 and ±17% for Group 2.

### Retrospective cohort, long-term evaluation

This cohort included samples from a retrospective observational study that originally included 22 BALF samples from patients with pulmonary aspergillosis. The samples confirmed invasive pulmonary aspergillosis (IPA) or chronic pulmonary aspergillosis (CPA).^5^ From these specimens, 13 BALF samples with positive Ptx3 results ranging from 1307 pg/ml to 5000 pg/ml when tested in 2018–2020 were selected. These samples were stored at −80°C and retested in January 2023.

### Statistical analysis

A comparative analysis of the blood serum and BALF Ptx3 concentration values obtained from the initial samples and those subjected to cryopreservation was performed using GraphPad Prism 10.2.3 (GraphPad, San Diego, CA, USA). The Wilcoxon signed-rank test was supplemented with analysis of variance (anova) and the Bonferroni test for statistical significance, comparing the T0–T4 Ptx3 storage points. Furthermore, linear regression analysis was performed to evaluate the correlation between the Ptx3 concentrations in the initial and frozen samples. Results were considered significant if*P* < .05.

## Results

**Figure 2.**
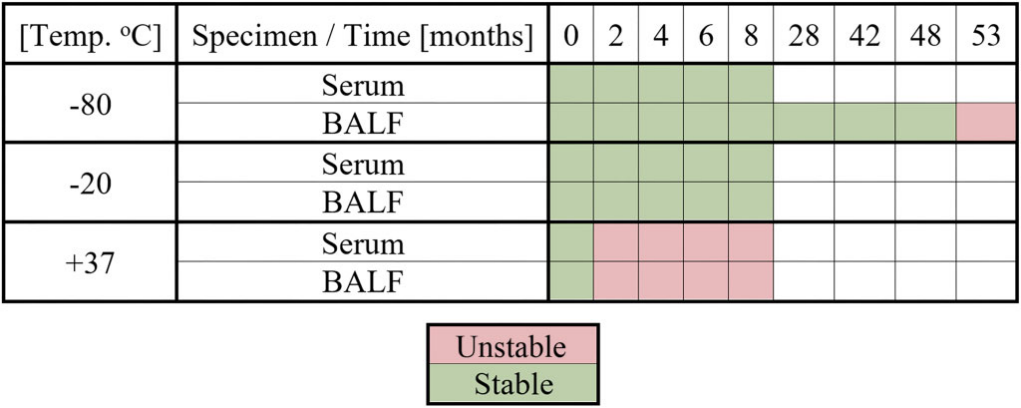
General overview of the stability of Pentraxin 3 in serum and BALF samples at different storage temperatures. Criteria for considering Ptx3 levels “stable” vs. “unstable” across the different conditions tested, the deviation in comparison to baseline at T0 was ±5% for Group 1 and ±7% for Group 2, maximally.

Ptx3 exhibited stability in both serum and BALF samples for 8 months when stored at −20 and −80°C. Furthermore, long-term storage at −80°C demonstrated stability for up to 48 months. In contrast, the samples kept at 37°C maintained stability only at the initial measurement point, as illustrated in Figure 2. An investigation of Ptx3 stability revealed variations over time in both serum and BALF specimens stored at various temperatures.

### Prospective cohort, real-time evaluation

#### Group 1

Ptx3 concentrations in the blood serum remained relatively stable at temperatures of −80°C and −20°C when compared to the initial concentration values (3015 and 3019 pg/ml for medium-positive and 4996 and 4956 pg/ml for high-positive pooled samples), as shown in Table 1. All samples were assessed prior to storage, followed by evaluations conducted at the second, sixth, and eighth months. During the storage period, the concentration varied slightly from the starting level, fluctuating between −0.8 and +1.3%. However, when stored at +37°C, the concentration of Ptx3 in medium-positive (3021 pg/ml) and high-positive (4990 pg/ml) serum samples apparently increased after 2 months of storage and remained elevated until the final measurement at 8 months. This increase was particularly notable in the medium-positive samples, where it reached up to 60.7%

**Table 1.** Real-time stability of pooled specimens at −80°C, −20°C, and +37°C.

Specimen type, storage	Ptx3 sample concentration (pg/ml) (% change^a^)				
temperature	0 m^T0^	2 m^T1^	4 m^T2^	6 m^T3^	8 m^T4^
**Serum, medium-positive**					
*−80*	3019	3002 (−0.6)	2999 (−0.7)	3003 (−0.5)	3015 (−0.1)
*−20*	3015	3024 (+0.3)	3054 (+1.3)	3012 (−0.1)	3002 (−0.4)
*+37*	3021	4854 (+60.7)	4258 (+40.9)	4125 (+36.5)	4420 (+46.3)
**Serum, high-positive**					
*−80*	4956	5000 (+0.9)	4915 (−0.8)	5002 (+0.9)	4986 (+0.6)
*−20*	4996	4991 (−0.1)	4999 (+0.1)	5001 (+0.1)	4990 (−0.1)
*+37*	4990	5068 (+1.6)	5254 (+5.3)	5321 (+6.6)	5049 (+1.2)
**BALF, medium-positive**					
*−80*	3056	3012 (−1.4)	3052 (−0.13)	3000 (−1.8)	3005 (−1.7)
*−20*	3012	3059 (+1.6)	3095 (+2.8)	3045 (+1.1)	3018 (+0.2)
*+37*	3001	289 (−90.4)	189 (−93.7)	145 (−95.2)	150 (−95)
**BALF, high-positive**					
*−80*	5026	4996 (−0.6)	5011 (−0.3)	5056 (+0.6)	5001 (−0.5)
*−20*	5089	4998 (−1.8)	5158 (+1.4)	5025 (−1.3)	5011 (−1.5)
*+37*	5013	356 (−92.9)	258 (−94.9)	296 (−94.1)	150 (−97)

a Relative to the initial 0 m^T0^ (starting point/month) Ptx3 concentration

For BALF samples, there was no variation in the concentration of Ptx3 at storage temperatures of −80°C and −20°C, and the concentrations in the medium (3056 and 3012 pg/ml) and high-positive (5026 and 5089 pg/ml) pooled samples remained consistent over a period of 8 months, with only minor fluctuations (−1.8% to +2.8%). In contrast, when stored at +37°C, the initial Ptx3 concentration in medium (3001 pg/ml) and highly positive (5013 pg/ml) BALF samples dropped significantly by the second measurement at 2 months (dropping to 289 and 356 pg/ml, respectively) and remained low until the final measurement at 8 months, reaching only 150 pg/ml in both cases.

#### Group 2

The second group, comprising 42 individual serum and BALF specimens, underwent real-time stability testing for Ptx3 at various temperatures (−80°C, −20°C, and +37°C) and over multiple time points (T0–T4). The detailed results of this test are presented in the Supplementary material (S1). The Ptx3 concentrations in both serum and BALF were similar to those observed in Group 1 (pooled samples), with average values remaining stable when stored at −80°C and −20°C for the entire 8-month period. However, at +37°C, Ptx3 levels in the serum increased over time, whereas Ptx3 levels in BALF samples quickly decreased.

Partial results comparing the percentage differences between the temperatures over time based on the original dataset (S1) are provided in Supplementary material (S2). The results revealed distinct patterns of changes across different storage temperatures for both the serum and BALF samples. In serum stored at −80°C, Ptx3 concentration at most time points was significantly higher than that at the initial time point (T0), with percentage changes ranging from +3.9% to +23.1%. Serum at −20°C showed statistically significant differences at all time points compared to T0, with % changes between +6.1% and +9.4%. In serum stored at +37°C, all time points exhibited statistically significant differences from T0 (*P* < .01), with substantial percentage changes ranging from +40.7% to +48.3%, indicating a notable apparent increase in the Ptx3 concentration.

**Figure 3.**
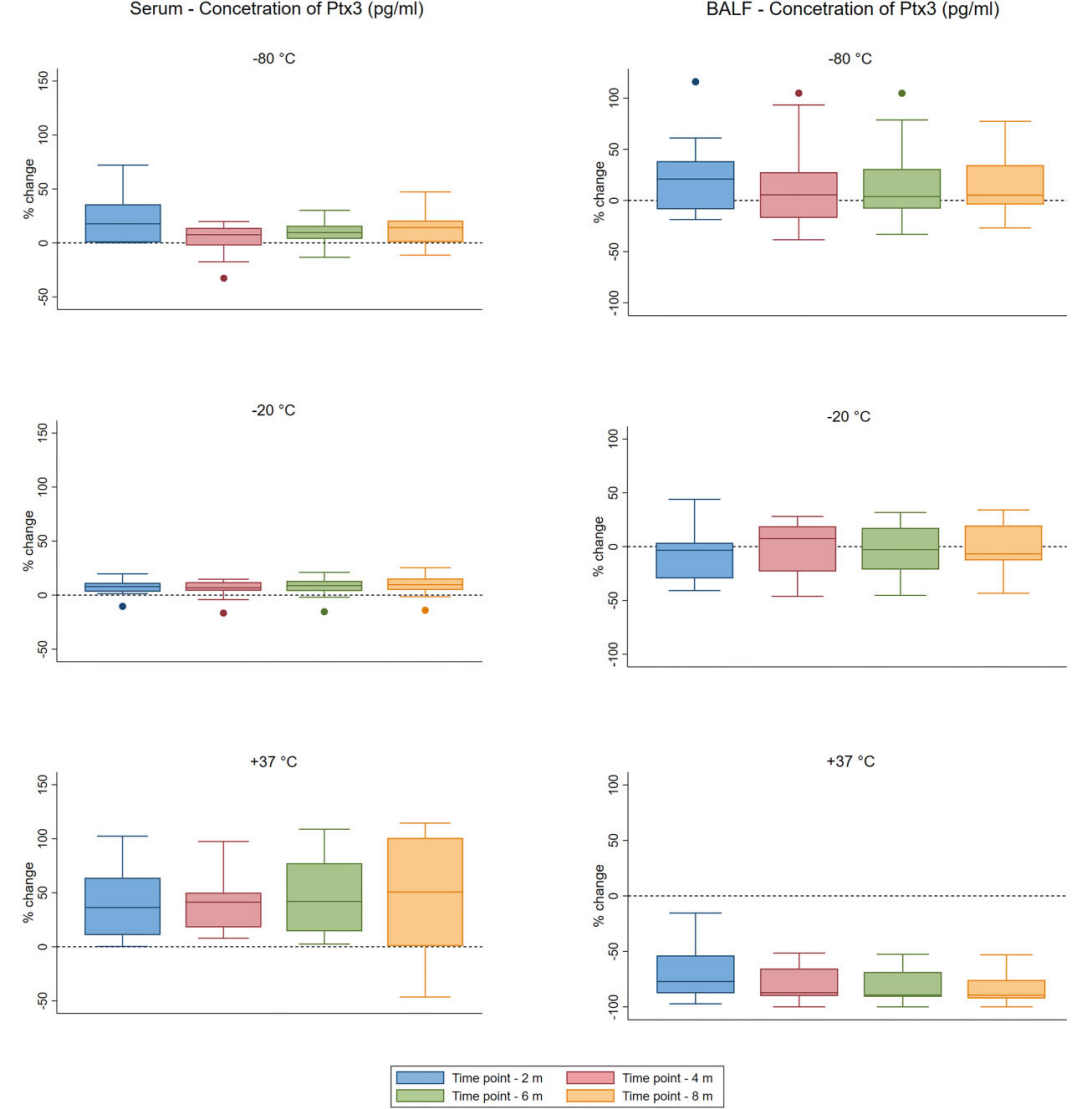
Real-time stability of 42 individual specimens at −80, −20, and +37°C. The concentration of Pentraxin 3 at time point 0, compared to its concentration at the time point indicated on the *x*-axis, represents the percentage change over time. Statistical analysis of the examined and re-examined measurements involved the Wilcoxon signed-rank test supplemented by analysis of variance (anova).

BALF samples stored at −80°C and −20°C were significantly different from T0, with higher variability noted based on the standard deviation. At +37°C, BALF Ptx3 showed statistically significant differences at all time points compared to T0 (*P* < .001), with percentage changes ranging from −84.0% to −67.9%, indicating a decrease in values. A comparison of the median temperatures is shown in Figure 3.

**Figure 4.**
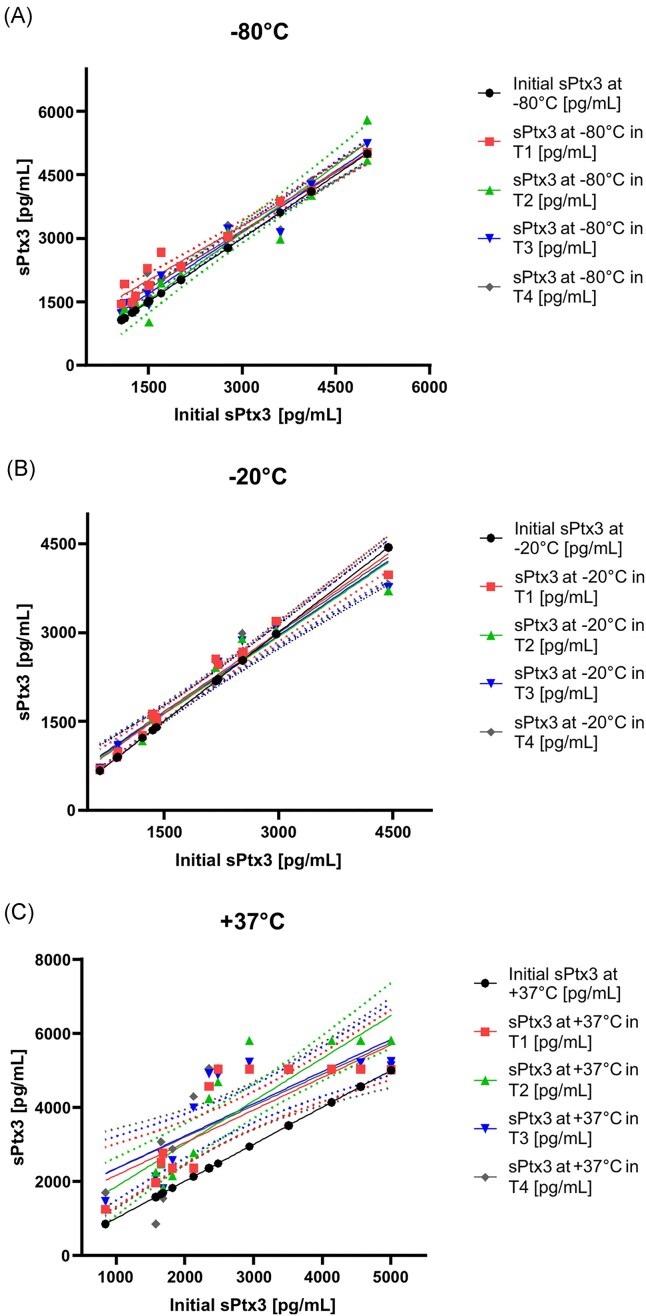
Linear regression showing the temperature dependence between Ptx3 determined from the patient’s initial samples (T0) and the individual serum samples (T1–T4) at −80°C, −20°C, and +37°C. The *y*-axis represents the mean Ptx3 level (pg/ml), as determined from the samples. Dashed lines represent 95% confidence intervals. BALF Ptx3, Pentraxin 3 in bronchoalveolar lavage fluid; T0–T4, time point of measurement.

Linear regression analysis was used to assess the goodness of fit between Ptx3 concentrations measured in the original and stored samples. For the blood serum specimens, the coefficient of determination (*R*^2^) between the initial Ptx3 concentration and those stored at −80°C and −20°C for 8 months were 0.9647 and 0.9724, respectively (*P* < .0001), indicating a strong correlation and excellent stability. This observation was further supported by the fact that all data points (T0–T4) were within the 95% confidence interval (Fig. 4A and B).

In contrast, the correlation between the initial Ptx3 concentration and the concentration after 8 months of storage at +37°C was calculated as *R*^2^ = 0.6145 (*P* = .0218) (Fig. 4C).

**Figure 5.**
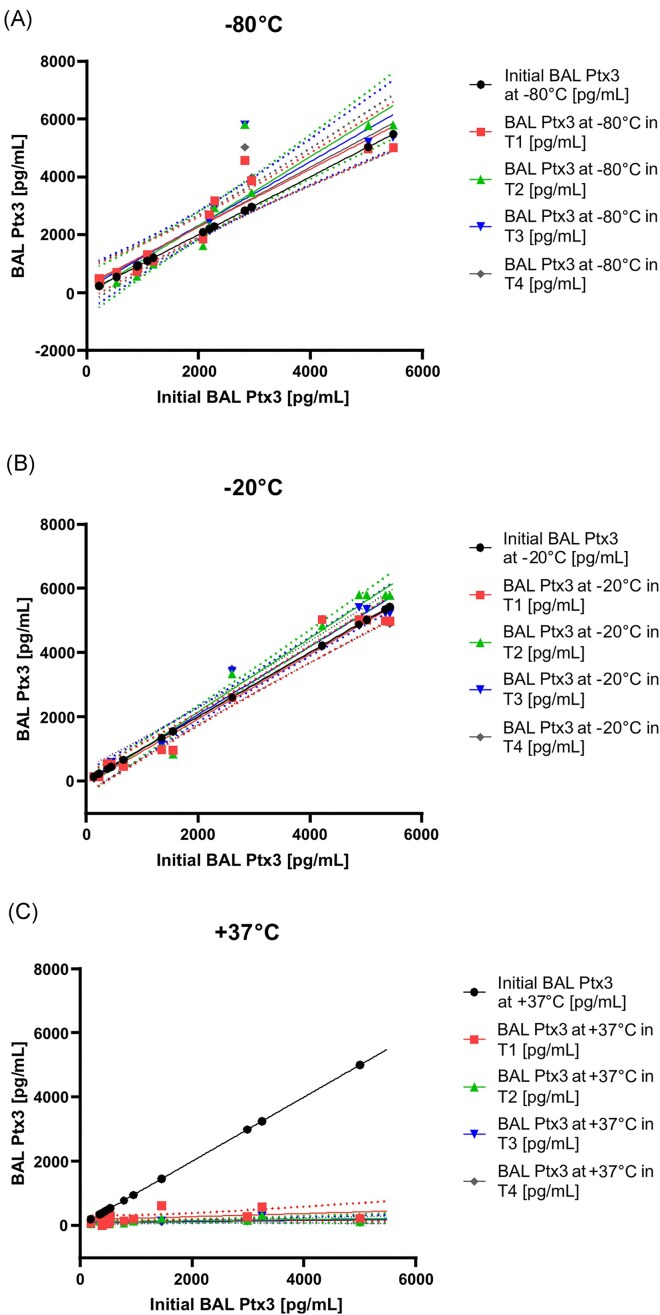
Linear regression showing the temperature dependence between Ptx3 determined from the patient's initial samples (T0) and individual frozen samples of BALF (T1–T4) at −80°C, −20°C, and +37°C. The *y*-axis represents the mean Ptx3 level (pg/ml) determined from frozen samples. Dashed lines represent 95% confidence intervals. BALF, bronchoalveolar lavage fluid; Ptx3, Pentraxin 3; T0–T4, time point of measurement.

For BALF specimens, all data points (T1–T4) were within the 95% confidence interval. Comparison of Ptx3 concentrations in the initial samples with those stored at −80°C and −20°C for 8 months showed *R*^2^ of 0.9692 and 0.9121, respectively (*P* < .0001) (Fig. 5A and B). In contrast to the blood serum samples, there was a significant difference in the mean Ptx3 concentration in BALF samples stored at +37°C for 8 months (1271 pg/ml vs. 109 pg/ml). The correlation between the Ptx3 concentration in the samples assessed at the initial stage and after 8 months of storage at +37°C was determined to be *R*^2^ = 0.3916, *P* = .1662 (Fig. 5C).

### Retrospective cohort, long-term storage evaluation

**Figure 6.**
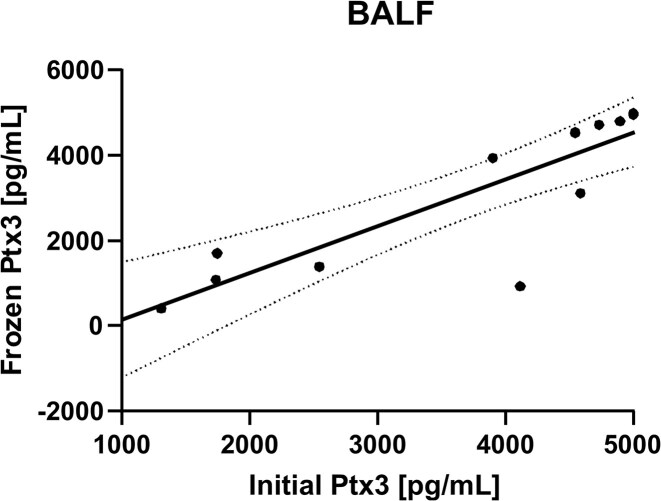
Linear regression showing the correlation between Ptx3 determined from the patient’s initial samples and frozen samples of BAL fluid. The *y*-axis represents the mean Ptx3 level (pg/ml) determined from frozen samples. The dashed lines represent the 95% confidence interval *R*^2^ = 0.8941.

Table 2 demonstrates the long-term stability of Ptx3 concentration in individual BALF specimens stored at −80°C. Ptx3 concentration before and after 48 months showed a significant difference in percentage change (*P* = .0016). During the first period (28–48 months), eight observations showed a mean percentage change of −0.67% ± 1.03%, ranging from −2.6% to 0.1%. In contrast, the second period (53–56 months) exhibited a more substantial decrease, with five observations showing a mean percentage change of −52.28% ± 19.87%, ranging from −77.4% to −32%. Linear regression analysis estimated *R*^2^ for the comparison between Ptx3 concentrations measured during the initial evaluation and those measured after freezing and storage to be 0.8941 (*P* < .0001), with 85% (11 of 13) of the data points within the 95% confidence interval (Fig. 6). The stability of Ptx3 concentrations in BALF samples ceased after 53 months of storage at −80°C (Table 2). These findings suggest that while the Ptx3 concentration in BALF specimens remained relatively stable during the first period, with only a slight decrease, long-term storage at −80°C may affect the stability of Ptx3 and cause a significant reduction in Ptx3 concentration, particularly after extended periods.

**Table 2.** Long-term stability of individual BALF specimens at −80°C.

Initial Ptx3 concentration (pg/ml)	No. of months frozen	Frozen Ptx3 (pg/ml)	% difference^a^
4544	28	4536	−0.2
3902	29	3940	+0.1
5000	43	4998	−0.04
5000	44	4955	−0.1
4733	45	4722	−0.2
1746	45	1701	−2.6
4897	46	4800	-2
4547	48	4535	−0.3
4588	53	3115	-32
1736	54	1084	-38
2545	55	1389	-45
1307	55	401	-69
4116	56	932	-77.4

a Comparison of initial Ptx3 BALF with frozen Ptx3 concentrations.

## Discussion

The implementation of Ptx3 in routine diagnostics requires standardized sample handling and storage conditions to guarantee the accurate reading and interpretation of results. This study demonstrated that Ptx3 levels in serum and BALF samples were stable over time under various temperature conditions, excluding +37°C, suggesting its potential utility as a reliable biomarker for disease diagnosis and monitoring in both retrospective and prospective clinical studies. The decision to investigate the stability of Ptx3 at 37°C was based on its relevance to specific laboratory procedures and applications. In numerous experimental protocols, including enzymatic reactions and cell culture experiments, sample incubation at 37°C is the standard practice to simulate physiological conditions. Elucidating Ptx3 stability under such circumstances is crucial for determining the suitability of pretreated or incubated samples for subsequent Ptx3 analysis. Our findings demonstrated that specimens maintained at 37°C for extended durations could not be reliably quantified for Ptx3 using commercially available IVDR kits. The results presented herein underscore the necessity of selecting optimal storage and handling conditions based on the specific biomarker analysis. The Ptx3 medium-positive and high-positive serum and BALF pools, as well as individual samples, showed minimal changes, with slight changes in Ptx3 concentrations ranging from −1.8% to 2.8%. Our data suggest that Ptx3 maintains its stability when subjected to freezing temperatures of −80°C and −20°C. Additionally, when stored at −80°C for extended periods of time, the concentration of Ptx3 samples remained consistent for up to 53 months following their initial measurement, after which the measured concentration began to rapidly decrease, falling within the range of 32%–77.4%. However, Ptx3 stability during storage at 37°C varied, with apparent increases in Ptx3 concentration ranging from 36.5% to 60.7% in serum and decreases from 92.9% to 97% in BALF samples. In fact, Ptx3 stored at 37°C has a higher binding capacity, causing an increase in Ptx3 concentration in the EIA reaction. Elevated temperatures can lead to protein degradation in BALF samples owing to the presence of various proteases originating from the present microbes. In the case of serum samples, long-term storage could lead to protein degradation, aggregation, or cryoprecipitation, which could affect the apparent ELISA-determined concentration due to several factors, such as increased avidity of interaction and exposure of additional binding sites, particularly in the case of polyclonal antibodies used in our ELISA assay, as well as oligomerization-caused signal loss, as was reported for amyloid proteins.^14^ Future research could address this issue through parallel measurement of Ptx3 concentrations, for example, by employing EIA tests coated with C1q binding circulating immune complexes or galactosaminogalactan^3^ and polyclonal anti-Ptx3 antibodies. However, for clinical applications, this consideration is not relevant, and the previously stated conclusion remains valid: BALF samples should not be analyzed using the available IVDR kits after prolonged storage at elevated temperatures (37°C).

We assumed that the contrasting behavior of Ptx3 concentrations in serum and BALF during storage at 37°C may be related to differences in protein stability and environmental factors. This decrease in BALF may have been caused by protein degradation or aggregation in this specific environment. Generally, the stability of proteins during freeze-thaw cycles can vary depending on their glycosylation status, with proteins that are highly N-glycosylated and exhibit greater stability. Factors such as pH, salt concentration, and temperature fluctuations can influence protein denaturation and activity loss during these processes.^15,16^ The freezing and thawing process can modify protein structures, as observed with CRP, a protein similar to Ptx3 that is released during the course of infection. This alteration may disrupt the pentameric form of CRP, causing it to break down into separate monomers. These monomers may be detected during analysis, which could lead to an overestimation of CRP levels in samples.^17^

The limited understanding of Ptx3 in biological fluids highlights the need for comprehensive research to improve our understanding of its characteristics. This would enhance the interpretation of Ptx3 findings in human samples and facilitate its application as a biomarker of infectious diseases in clinical settings.

### Strengths and limitations

All tests were conducted using a single lot of the BioVendor Ptx3 EIA kit, which does not account for the potential lot-to-lot variability. Additionally, a higher number of examined samples in both prospective and retrospective cohorts, including patient populations in various clinical scenarios, would be beneficial to improve the strength of the study. Conducting an analysis of several technical replicates for each pool would have yielded more reliable data and enabled broader conclusions for Group 1. The choice to use single aliquots was driven mainly by practical limitations regarding sample availability and the necessity to preserve scarce clinical specimens. Furthermore, for this type of biomarker, our goal was to replicate real-world clinical laboratory procedures in which only one measurement is typically performed. In the context of blood serum samples, it is also important to note that the practical application of Ptx3 focuses on daily consecutive use, which involves repeated measurements of new samples each day. To manage potential variability, we included a larger number of individual samples in Group 2 (42 serum and BALF samples) that were examined at various time points. The alignment between the pooled (Group 1) and individual (Group 2) sample outcomes reinforces confidence in the overall conclusions of the study.

This study limited to storage temperatures at −80°C, −20°C, and +37°C in serum and BALF samples with medium and high Ptx3 concentrations, but other potentially relevant temperatures (e.g., room temperature or refrigeration at 4°C), biological fluids (e.g., plasma and urine), and concentrations (e.g., low and near-cutoff concentrations) may be of interest. To enhance the complexity of understanding the Ptx3 biomarker, other factors such as multiple freeze-thaw cycles, a comparison with freshly collected samples at each selected time point, pH, presence of proteases, and other matrix factors may be considered in future studies to provide an additional context for the stability findings.

This study demonstrated that Ptx3 concentrations remain relatively stable when stored at −80°C and −20°C for up to 8 months in both serum and BALF samples. Long-term stability was observed in BALF samples stored at −80°C for up to 53 months, after which the concentration began to decrease significantly. Storage at 37°C resulted in divergent effects; serum samples showed increased Ptx3 concentrations, whereas BALF samples exhibited substantial decreases. Further research is required to characterize these observations at a molecular level.

## Supplementary Material

myaf057_Supplemental_Files

## Data Availability

The data underlying this article are available in the article and in its online supplementary material.
