# Supplementary material for: Time- and temperature-dependent Pentraxin 3 stability in serum and bronchoalveolar lavage fluid samples
Source: Med Mycol. 2025 Jun 27;63(7):myaf057. doi: 10.1093/mmy/myaf057 (PMC12247167; doi:10.1093/mmy/myaf057)
Supplement: myaf057_Supplemental_Files [file myaf057_supplemental_files.zip › mm-2025-0010-File009.docx]

|  |  | Concentration of Ptx 3 (pg/ml) - **% change** | | | | | | | |
| --- | --- | --- | --- | --- | --- | --- | --- | --- | --- |
| Storage temperature |  | Serum | | | | BALF | | | |
|  |  | TP 1 | TP 2 | TP 3 | TP 4 | TP 1 | TP 2 | TP 3 | TP 4 |
| T1: -80 °C | mean±sd | 23.1±23.70 | 3.9±14.59 | 9.4±11.69 | 13.9±15.03 | 22.7±37.02 | 12.9±42.51 | 17.5±38.37 | 16.0±28.99 |
|  | p-value* | (0.001) | (0.198) | (0.026) | (0.011) | (0.158) | (0.221) | (0.246) | (0.177) |
| T2: -20 °C | mean±sd | 7.1±7.46 | 6.1±8.43 | 7.6±8.81 | 9.4±9.79 | -6.9±24.43 | -0.7±25.78 | -2.0±24.86 | -2.9±25.54 |
|  | p-value* | (0.016) | (0.022) | (0.019) | (0.019) | (0.397) | (0.124) | (0.594) | (0.975) |
| T2: +37 °C | mean±sd | 40.7±32.89 | 43.3±28.05 | 48.3±36.32 | 46.9±51.05 | -67.9±24.59 | -80.2±16.63 | -82.4±14.72 | -84.0±13.83 |
|  | p-value* | (0.001) | (0.001) | (0.001) | (0.005) | (0.001) | (0.001) | (0.001) | (0.001) |
| Comparison by Temperature (p-value)+ | T1xT2 | 0.253 | 1 | 1 | 1 | 1 | 0.033 | 0.735 | 0.211 |
|  | T1xT3 | 0.171 | <0.001 | <0.001 | <0.001 | 0.024 | <0.001 | <0.001 | <0.001 |
|  | T2xT3 | 0.002 | <0.001 | <0.001 | <0.001 | 0.009 | <0.001 | <0.001 | <0.001 |

% change TPx = (value TPx - value TP0)/value TP0*100, TP - Time point, x = 0 (0 m), 1 (2 m), 2 (4 m), 3 (6 m), 4 (8 m)

*comparison between value TP0 and TPx, Wilcoxon signed-rank test

+ analysis of variance (ANOVA) and Bonferroni test
